# Eccentric indices based QSPR evaluation of drugs for schizophrenia treatment

**DOI:** 10.1016/j.heliyon.2025.e42222

**Published:** 2025-01-23

**Authors:** Muneeba Mansha, Sarfraz Ahmad, Zahid Raza

**Affiliations:** a COMSATS University Islamabad, Lahore Campus, Pakistan; b Department of Mathematics, College of Sciences, University of Sharjah, United Arab Emirates

**Keywords:** Molecular graph, Eccentric indices, Regression models, Correlation coefficients, QSPR analysis, Statistical analysis

## Abstract

Schizophrenia is a long-term, serious mental health condition that affects how a person thinks, perceives, and behaves. This disorder often results in substantial difficulties in social interactions and work performance. Individuals with schizophrenia might appear disconnected from reality, causing significant distress both for themselves and their Friends. Although symptoms of schizophrenia can vary from person to person, they typically fall into three main categories: cognitive, negative, and psychotic. Creating computational tools to find and develop drugs for schizophrenia has more interest in the past few years. Regardless of the significant developments in drug design, the fundamental approach still uses topological descriptors. Topological indices are used to estimate the bioactivity of chemical compounds in QSAR/QSPR studies. In general, with the use of the quantitative structure-property relationship (QSPR), topological indices are numerical values that are connected to the chemical drug structures and are used to predict their reactivity, stability, and properties. This work focuses on calculating different eccentric indices (EIs), developing a regression model for thirteen anti-schizophrenia drugs, and applying statistical methods to establish a linear regression relationship between QSPR correlating properties and eccentric indices. Statistical analysis shows that p-values less than equals 0.05, f-test value (>2.5), and values of correlation *r* are greater than 0.7 validate the calculations. The correlation coefficient $\left(r^{2}\right)$ is a convenient tool for evaluating the QSPR models' quality. $r^{2}>0.7$ is essential for a good QSPR model. The p-values show the significance of the results, while the correlation coefficient values show the accuracy of the results. In order to fit regression models for the calculated eccentric index values, eight physicochemical properties of anti-schizophrenia drugs are examined. Drug properties like molar refractivity (cm^3^), refractive index (cm^3^), enthalpy (kJ/mol), melting, boiling and flash points (°C), complexity, and molecular weight are all more effectively estimated by the QSPR model. By examining actual and estimated values for the drugs, the results are verified.

## 1 Introduction

Schizophrenia is a serious mental illness that affects how a person thinks, feels, and behaves [1]. Treatments for schizophrenia nowadays concentrate on assisting patients to manage their symptoms, enhancing daily functioning, and accomplishing personal objectives like finishing school, pursuing a career, and maintaining happy relationships. One percent of people worldwide suffer from schizophrenia, a destructive mental illness. Men and women are equally affected, although women typically experience symptoms later than men. Paranoid, disorganized, catatonic, undifferentiated, and residual are the five forms of schizophrenia [2], [3]. Positive and negative symptoms are characteristics of schizophrenia [4], [5]. Hallucinations, voices that talk to or about the patient, and frequently paranoid delusions are examples of positive symptoms. Flattened affect, loss of pleasure, lack of will or drive, and social disengagement are examples of negative symptoms. It was discovered that the most prevalent first-rank symptom belonged to thought disorders [6]. Most schizophrenic individuals do not act violently. In general, the likelihood of suffering harm from others is higher for those who have schizophrenia than for those who do not. Dissociative identity disorder is characterized by the presence of two or more separate identities, each with unique behaviors and memories. A family history of schizophrenia is possible. Patients can talk to a primary care physician about concerns regarding their mental health. Alcohol and drug abuse are common issues for people with schizophrenia. Treatment for substance abuse and schizophrenia should be combined because treatment for one condition can impede treatment for the other [7], [8]. An estimated 5% to 6% of people with schizophrenia commit suicide, which is higher than average and typically happens in the months or years after the illness first manifests [9], [10]. Thirty to fifty percent of people with schizophrenia refuse to acknowledge their illness or follow their doctor's advice [11]. The Greek word schizein is the source of the Modern Latin word schizophrenia, which means “splitting of the mind” [12]. The DSM II was a broad, clinically based diagnostic model for schizophrenia that was within use in the US in the early 1970s. Using the ICD-9 criteria, schizophrenia was diagnosed far more frequently in the US than in Europe [13]. Approximately 0.3% to 0.7% of people will experience schizophrenia at some point in their lives [14], [15]. Before the 1960s, women and non-violent small-time offenders were occasionally diagnosed with schizophrenia, with the latter group being stigmatized for failing to fulfill their patriarchal roles as mothers and wives [16]. There is variation in the frequency of schizophrenia worldwide [2], within nations [17], as well as locally and neighborhood-wide. This variation can reach five times in frequency across studies conducted over time, across geographic locations, and by gender [18], [19]. Gaining more insight into the underlying molecular mechanisms of schizophrenia is necessary for the development of novel and efficacious medications. In the area of discovering drugs and structures, computational techniques like quantitative structure-property relationship (QSPR) analysis have been increasingly effective in recent years. More recently, powerful and effective drugs for the disorder have been developed using several QSAR/QSPR models and other clinical procedures [20], [21], [22], [23]. To predict the biological activities and characteristics of various chemical compounds, it investigates quantitative structure-activity (QSAR) and structure-property (QSPR) connections. Topological indices and physicochemical characteristics were used in the QSAR/QSPR study to predict the chemical compounds' bioactivity [24]. Both linear and non-linear models can be analyzed using QSAR. In recent years, there has been a lot of interest in using graph invariant (topological indices) in QSPR and QSAR studies. The most significant application of topological indices lately is in the non-empirical Quantitative Structure-Property Relationships (QSPR) and Quantitative Structure-Activity Relationships (QSAR). However, they have been used in many fields of chemistry, physics, mathematics, informatics, biology, etc [25]. The main goal of QSTR, QSPR, and QSAR modeling are to find possible drugs for treatment by using the topological descriptor's prediction power to link molecular structures to chemical and physical characteristics. QSPR modeling offers significant insights for creating molecules with specific properties, such as those aimed at treating schizophrenia, even if it is not directly involved in drug development. It is an essential tool for improving the drug development process within the context of chemical graph theory. For more details about QSTR, QSPR, and QSAR analysis refer to studies [26], [27], [28]. With the use of current experimental data, machine learning (ML) algorithms can model quantitative structure-property relationships (QSPR) and predict the properties of novel molecules. ML models, known as quantitative structure-property relationship (QSPR), correlate molecular properties to compound structure data. A particular component of artificial intelligence (AI), which attempts to model human abilities like learning, reasoning, and problem-solving, is machine learning (ML). The fields of chemistry, mathematics, and pharmaceutical sciences have numerous other uses for AI and ML. When predicting the pharmacokinetic characteristics of novel compounds (drugs), the QSPR method is crucial. A molecule's physicochemical and structural characteristics must be expressed in QSAR models using numerical values called descriptors. To improve QSAR models and enable more precise predictions and a deeper comprehension of structure-activity correlations, machine learning (ML) has emerged as a useful approach. QSAR investigations are greatly improved by machine learning, which also offers strong drug discovery capabilities. For further study, see [29], [30], [31], [32].

In recent years, graph neural networks (GNNs) have gained a lot of interest in the processing and analysis of this type of graph-structured data. Using graph theory to model molecular structures as graphs, with atoms acting as nodes and bonds as edges, graph neural networks (GNNs) have become extremely effective tools in drug development. These networks learn the topological and chemical connections inside molecular graphs, allowing for the prediction of drug characteristics, activities, and interactions. Because graph neural networks (GNNs) use graph theory to define molecular structures and interactions effectively, they have completely changed computational drug design and repurposing. GNNs are essential for accelerating medications' computational design and optimization since their predictive accuracy is further increased by integrating them with topological indices. GNNs produce insightful embeddings of graph-structured data [33], [34], [35].

Molecules and molecular compounds are often seen as molecular networks, with vertices and edges in a molecule network replacing the compound's atoms and chemical bonds, respectively. Particularly in QSPR/QSAR studies, molecular descriptors are important in mathematical chemistry [36]. This research aims to advance our knowledge of the molecular mechanisms underlying antipsychotic drugs and to help create drugs that are more effective in treating schizophrenia [37], [38]. The structural analysis of chemical graphs that represent chemical systems is the primary objective of chemical graph theory, which uses computational data to determine chemical properties. A lot of work highlights the significant role molecular structure plays a role in determining pharmacological interactions relevant to; lately, research focuses on computational and applications of the topological indices for different drugs like anti-cancer, novel drugs used in cardiovascular and Schizophrenia disease treatment, see [39], [40], [41], [42], [43], [44].

QSAR, QSPR, and QSTR modeling is widely recognized and firmly established area of research This type of modeling involves combining physicochemical and molecular characteristics with a medication's bioassay intended to produce a typical pharmacological reaction. By modeling the property-based on chemical descriptors, the descriptor-based QSPR methods utilize the complete structure of compounds.

As a result, the most exact predictions are possible. A 1D descriptor is a linear representation in one dimension of a molecule, while 2D descriptors represent the molecule on a two-dimensional plane, and 3D descriptors represent it in three-dimensional space.

Mathematical models correlating different physical, biological, and chemical characteristics are known as quantitative structure-activity relationships (QSARs). Look at the Figure for a broad summary of this manuscript. This article aims to create a QSPR analysis for drugs used to treat schizophrenia:

• To start, we gathered information about 13 drugs employed in schizophrenia treatment, along with their eight physicochemical characteristics.
• Then, we compute the numeric values of different eccentric indices to examine the molecular configuration of 13 schizophrenia drugs.
• A correlation between the indices and properties was subsequently established using the QSPR analysis. We used two different regression expressions, linear and quadratic, to conduct this research. These models have been selected because they are important in developing the connection between indices and drug properties.
• One statistical parameter, the correlation coefficient (r), offers information about the dependability and importance of the connection between the numeric values and the properties of schizophrenia drugs.
• Graphical representation displays the correlations between the indices and drug properties. Bar and Line graphs visually compare numerical data of all correlation coefficients.

## 2 Definitions and results

A simple graph is denoted as G=[V(G),E(G)], where |V(G)| is the order and |E(G)| is the size of graph *G*. V(G) is the set of vertices and E(G) represents the set of edges. Every vertex within the graph corresponds to an atom, and an edge is represented by a connection between two atoms. The valency or degree of a vertex is the number of edges incident to that vertex. Here, we discuss some useful topological indices (TI's). A topological index is a numerical value linked to the chemical constitution of a substance. This value is used to establish a correlation between the chemical structure and its physical properties, chemical reactivity, or biological activity. In reality, topological indices are created by converting a molecular graph into a number that describes the topology of that graph.

A strong base for comprehending the evolution and uses of topological indices in molecular graph theory is offered by the following literature. The Wiener index, a novel distance-based descriptor that is frequently used for evaluating molecular structures, was first presented by Gutman and Trinajstić (1972). In their 2014 research, Bora and Narasimha Murty examined the computational features of graph-theoretic indices and highlighted how useful they are for drug discovery. A thorough examination of degree-based indices was provided by Das and Trinajstić (2015), who also highlighted the adaptability of these indices in chemical applications. The importance of M-polynomials in calculating complex topological indices was further underlined by recent optimization developments, demonstrating its contemporary applicability in mathematical chemistry. For more details, see [45], [46], [47], [48].

The eccentricity of a vertex is an important idea within this framework, as it evaluates the maximum distance between 2 vertices. The distance between two vertices *u* and *v* in V(G) is the shortest *u*-*v* path length in *G*. The eccentricity of the vertex evaluates the maximum distance from a specific vertex to any other vertex in the graph. Formally, it can be expressed as:

ξ(v)=max{d(v,u)|∀u∈V(G)}

The total eccentricity index was introduced by Farooq et al. [49]. It is the sum of all the vertex eccentricities of graph *G*, which is defined as,

$$\zeta (G)=\sum_{v\in V(G)}\xi (v)$$

where eccentricity of vertex *v* is represented by ζ(G).

### 2.1 Average eccentricity

The average eccentricity avec(G) is the mean eccentricity value of all vertices in a graph *G* i.e.,

$$avec(G)=\frac{1}{n}\sum_{v\in V(G)}\xi (v)$$

A lot of work has been done for this eccentric index; see [50], [51].

### 2.2 Modified versions of Zagreb indices

The new modified versions of the Zagreb indices of a molecular graph *G* were proposed by D. Vukičević et al. in 2010 and Ghorbani et al. in 2012 [52], [53]. The following is an expression of modified versions of Zagreb indices in terms of eccentricity:

$$M_{1}^{⁎}(G)=\sum_{uv\in E(G)}[\xi (u)+\xi (v)]$$

The second Zagreb eccentricity index is defined as:

$$M_{1}^{⁎⁎}(G)=\sum_{v\in E(G)}{\left[\xi (v)\right]}^{2}$$

The third Zagreb eccentricity index is defined as:

(2.1) $$M_{2}^{⁎}(G)=\sum_{uv\in E(G)}\xi (u).\xi (v)]$$

For each u∈V(G), we observe that ξ(u) appears exactly d(u) times in the sum (2.1). Therefore

$$M_{1}^{⁎}(G)=\sum_{uv\in E(G)}\xi (u)+\xi (v)=\sum_{u\in E(G)}d(u)\xi (v)=\xi (G)$$

Thus it is insignificant to compute $M_{1}^{⁎}(G)$

### 2.3 Geometric-arithmetic index

The “eccentricity based geometric-arithmetic (GA)” index of a graph *G* is defined as [54],

$$GA_{4}(G)=\sum_{uv\in E(G)}\frac{2\sqrt{\xi (u).\xi (v)}}{\xi (u)+\xi (v)}$$

Additional findings about the geometric-arithmetic index based on eccentricity and the average eccentricity index are found in [55].

### 2.4 Atom-bound connected based on eccentricity index

A new version of the *ABC* index is introduced by Farahani [56] as,

$$ABC(G)=\sum_{uv\in E(G)}\frac{\sqrt{\xi (u)+\xi (v)-2}}{\xi (u).\xi (v)}$$

The eccentricity-based geometric-arithmetic index and the eccentricity-based ABC index for copper oxide were calculated by Imran et al. in [57]. The eccentric ABC index of a linear polycene parallelogram benzenoid was computed by Gao et al. [58]. Another remarkable topological descriptor is the harmonic index [59], defined in Fajtlowicz (1987) as,

$$H(G)=\sum_{uv\in E(G)}\frac{2}{d(u)+d(v)}$$

This index has attracted great interest in the last years (see, e.g. Deng, Balachandran, Ayyaswamy, & Venkatakrishnan, 2013 [60]; Li & Shiu cite, 2014; Rodriguez & Sigarreta, 2017 [61]; Shwetha Shetty, Lokesha, & Ranjini, 2015 [62]; Wu, Tang, & Deng, 2013 [63]; Zhong, 2012 [64]). In particular, in Shwetha Shetty et al. (2015) [62] it appears relations for the harmonic index of some operations of graphs.

### 2.5 Eccentric harmonic index of a graph

Let *G* be a graph consisting of m edges and n vertices. Then, we define the eccentric harmonic index $H_{e}(G)$ of *G* as follows:

$$H_{e}(G)=\sum_{uv\in E(G)}\frac{2}{\xi (u)+\xi (v)}$$

**Figure 1:**
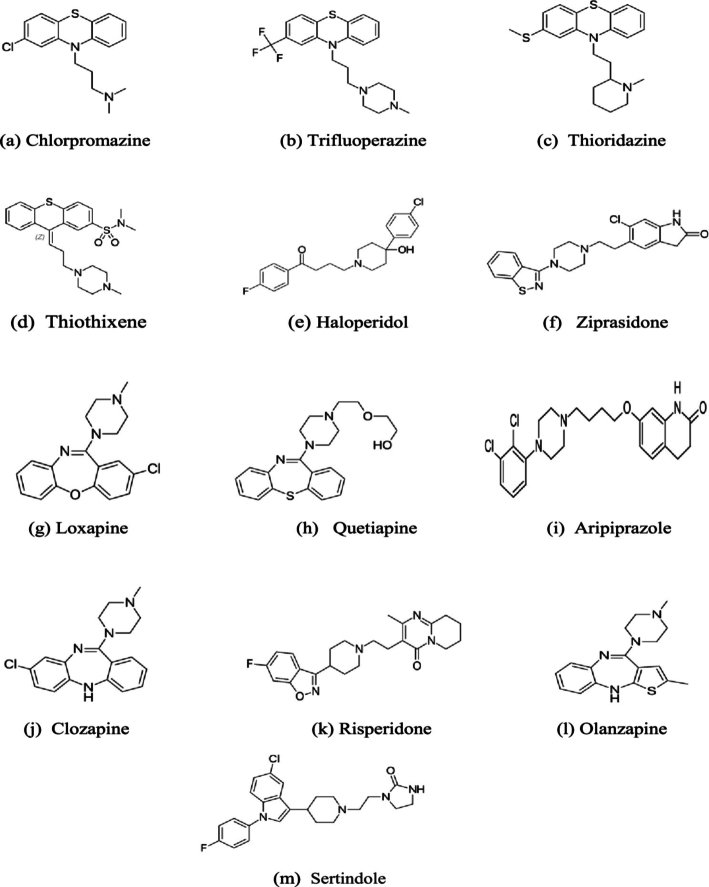
Drugs for Schizophrenia Treatment.

These indices are calculated for the drugs listed in Fig. 1. Some of the drugs used to treat schizophrenia have strong correlations among their physical and chemical features and the topological indices outlined above, which are shown by QSPR analysis. Fig. 1 shows the molecular structures of drugs to treat schizophrenia. Chemists and pharmacists utilize drug-related data, such as boiling point (BP), melting point (MP), enthalpy (E), flash point (FP), molar refractivity (MR), complexity (C), molecular weight (MW), and refractive index (R). The Edge and vertex partition method is employed to calculate the eccentric topological indices in a graph based on the degree distance. The indices are determined by the vertex degree and the maximum distance from a vertex to all other vertices. It considers the potential eccentric structures based on edges and distances between all edges and vertices. A vertex's degree in a graph indicates how many edges are connected to it.

$\begin{array}{cc} & E_{\left(2,2\right)}=\left\{e=uv\in E(G)|d_{u}=2,d_{v}=2\right\},\\ & E_{\left(2,3\right)}=\left\{e=uv\in E(G)|d_{u}=2,d_{v}=3\right\},\\ & E_{\left(3,3\right)}=\left\{e=uv\in E(G)|d_{u}=3,d_{v}=3\right\},\\ & E_{\left(1,3\right)}=\left\{e=uv\in E(G)|d_{u}=1,d_{v}=3\right\}, \end{array}$ $\begin{array}{cc} & E_{\left(1,4\right)}=\left\{e=uv\in E(G)|d_{u}=1,d_{v}=4\right\},\\ & E_{\left(3,4\right)}=\left\{e=uv\in E(G)|d_{u}=3,d_{v}=4\right\},\\ & E_{\left(1,2\right)}=\left\{e=uv\in E(G)|d_{u}=1,d_{v}=2\right\}\\ & E_{\left(4,2\right)}=\left\{e=uv\in E(G)|d_{u}=4,d_{v}=2\right\}. \end{array}$

$\begin{array}{cc} & e(v)=\left\{v\in V(G)|max\{d(u,v)\}=5\right\},\\ & e(v)=\left\{v\in V(G)|max\{d(u,v)\}=6\right\},\\ & e(v)=\left\{v\in V(G)|max\{d(u,v)\}=7\right\},\\ & e(v)=\left\{v\in V(G)|max\{d(u,v)\}=8\right\},\\ & e(v)=\left\{v\in V(G)|max\{d(u,v)\}=9\right\},\\ & e(v)=\left\{v\in V(G)|max\{d(u,v)\}=10\right\}, \end{array}$ $\begin{array}{cc} & e(v)=\left\{v\in V(G)|max\{d(u,v)\}=11\right\},\\ & e(v)=\left\{v\in V(G)|max\{d(u,v)\}=12\right\},\\ & e(v)=\left\{v\in V(G)|max\{d(u,v)\}=13\right\},\\ & e(v)=\left\{v\in V(G)|max\{d(u,v)\}=14\right\},\\ & e(v)=\left\{v\in V(G)|max\{d(u,v)\}=15\right\}.\\ & e(v)=\left\{v\in V(G)|max\{d(u,v)\}=16\right\}.\\ & e(v)=\left\{v\in V(G)|max\{d(u,v)\}=17\right\}. \end{array}$

**Figure 2:**
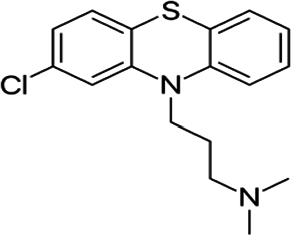
Chlorpromazine drug structure.

Boiling points must be used with the other drugs to treat schizophrenia. The boiling point (BP) molecular structure is shown in 2D in Fig. 2. There are 23 edges and 21 vertices in the molecular structure of BP. A method of distance-based eccentric indices has been used to evaluate this structure. The range of the vertex set of this structure BP is from 1 to 23. As will be discussed below, the thirteen different eccentricity-based topological indices of the BP structure are calculated using the distance-based vertex. The same methodology can be applied to determine the remaining indices of other drug structures.

$$RM_{1}(\text{BP})=\sum_{v\in V(G)}\xi (v)=5(9)+6(8)+5(7)+3(6)+2(5)=156$$

$$avec(G)=\frac{1}{n}\sum_{v\in V(G)}\xi (v)=156/21=7.4286$$

$$M_{1}^{⁎}(G)=\sum_{uv\in E(G)}[\xi (u)+\xi (v)]=1(5+5)+7(8+9)+7(7+8)+5(6+7)+3(6+5)=332$$

$$M_{1}^{⁎⁎}(G)=\sum_{v\in E(G)}{\left[\xi (v)\right]}^{2}=[{\left(5(9)\right]}^{2}+[{\left(6(8)\right]}^{2}+[{\left(5(7)\right]}^{2}+{\left[3(6)\right]}^{2}+{\left[2(5)\right]}^{2}=5978$$

$$M_{2}^{⁎}(G)=\sum_{uv\in E(G)}[\xi (u).\xi (v)]=[1][5\times 5]+[7][8\times 9]+[7][7\times 8]+[5][6\times 7]+[3][6\times 5]=25+7(72)+7(56)+5(42)+3(30)=1221$$

$$GA_{4}(G)=\sum_{uv\in E(G)}\frac{2\sqrt{\xi (u).\xi (v)}}{\xi (u)+\xi (v)}=\frac{2\sqrt{5\times 5}}{5+5}+7\left(\frac{2\sqrt{8\times 9}}{8+9}\right)+7\left(\frac{2\sqrt{7\times 8}}{7+8}\right)\;+5\left(\frac{2\sqrt{6\times 7}}{6+7}\right)+3\left(\frac{2\sqrt{6\times 5}}{6+5}\right),=22.9450$$

$$ABC(G)=\sum_{uv\in E(G)}\frac{\sqrt{\xi (u)+\xi (v)-2}}{\xi (u).\xi (v)}=\frac{\sqrt{5+5-2}}{5\times 5}+7\left(\frac{\sqrt{8+9-2}}{8\times 9}\right)+7\left(\frac{\sqrt{7+8-2}}{7\times 8}\right)\;+5\left(\frac{\sqrt{6+7-2}}{6\times 7}\right)+3\left(\frac{\sqrt{5+6-2}}{5\times 6}\right)=\frac{2\sqrt{2}}{25}+\frac{7\sqrt{15}}{72}+\frac{\sqrt{13}}{8}+\frac{5\sqrt{11}}{42}+\frac{3}{10}=1.6352$$

$$H_{e}(G)=\sum_{uv\in E(G)}\frac{2}{\xi (u)+\xi (v)}\;\frac{2}{5+5}+7\left(\frac{2}{8+9}\right)+7\left(\frac{2}{7+8}\right)+5\left(\frac{2}{6+7}\right)+3\left(\frac{2}{6+5}\right)=3.2715$$

Mathematical formulas that consider the degrees of vertices in a graph are used to calculate degree-based topological indices. We collected data on the physiochemical properties of drugs used for treating schizophrenia from the PubChem website. We obtained the results using techniques involving vertex degree, distance between vertices, and edge division to determine the numerical values of molecular descriptors. Eight statistical parameters (p-value and f-test value) and two types of regression equations, linear and quadratic, are utilized for the QSPR analysis. The SPSS software calculates the correlation results for these two regression models. Chemical drawing software applications such as Chem-Draw and Chem-Doodle are used to create graphic representations of these drugs' 2D molecular structures. These graphs are made using MS Excel. Matlab can be used to compute all indices, while Maple is specifically used for graph plotting. This approach offers the benefit of quickly calculating all indices. This manuscript provides clear and understandable work and literature for readers.

## 3 Regression models

The eccentric indices are utilized in the modeling of specific significant physical properties, including BP, MP, E, FP, MR, RI, C, MW, and R of 13 drugs constructed in Fig. 1 for the treatment of schizophrenia. The calculated values of the physical properties of different anti-schizophrenia drugs are provided in Table 1. Regression models are valuable statistical instruments used for analyzing data and making predictions. This analysis examines linear and quadratic regression models. Linear regression is a straightforward technique designed to effectively depict the connection between a dependent variable (Y) and an independent variable (EI). So, an equation for the linear regression is defined as:

Y=α+β(EI)

Eccentricity indices and molecular properties are found to be directly proportionately correlated using linear regression models. Here, eccentricity can be an independent variable to predict molecular properties linearly. In the context of QSPR studies, linear regression often shows how changes in the eccentricity index directly correlate with changes in the property of interest. The significance of eccentricity in linear regression is often validated through correlation coefficients (*r*) and p-values. For instance, high positive correlations (*r* values close to 1) indicate that eccentricity is a strong predictor of the property. Quadratic regression is a statistical technique for analyzing curved relationships between dependent (Y) and independent variables (EIs). The model consists of a y-intercept (*α*), a linear slope (*β*), and a quadratic term (*γ*). The following is the expression for the quadratic regression equation:

$$Y=\alpha +\beta (EI)+\gamma {\left(EI\right)}^{2}$$

The efficiency of quadratic regression is evaluated by examining the coefficient of determination $\left(r^{2}\right)$ and f-values, which indicate how well the model explains the variability in the data. By examining the chemical structure of materials and chemicals, the QSPR (Quantitative Structure-Property Relationship) technique makes predictions about their behaviors and properties. Statistical parameters are employed to find the most suitable model that represents the relationship between the structure and property. The accuracy and dependability of the model are evaluated by using many parameters, like the correlation coefficient *r* and $r^{2}$, p-value, f-value, and model performance. This research focuses on analyzing statistical properties for eccentric topological indices in two QSPR regression models, linear and quadratic. The statistical analysis used in the QSPR study of eccentric indices is shown in Table 3, Table 4, Table 5, Table 6, Table 7, Table 8, Table 9, Table 10. Furthermore, Table 11, Table 12 present a comparison between the computed and actual values of the model, providing useful information regarding data values. The dataset size, 9, 12, or 13, is represented by the “*n*” variable. Three possible conditions exist in the correlation (*r*) that is r>0 to show a strong or consistent relationship, r<0 to show a negative or inverse relationship, and r=0 to show no relation between the two variables. The inverse correlation value lies between −0.1 and −0.6. The correlation coefficient *r* ranges from −1 to +1. A value of +1 represents a perfect positive correlation, whereas a value of −1 represents a perfect negative correlation. An *r* value greater than 0.8 is generally considered a strong correlation in QSPR for drug properties and shows a weak correlation with (r<0.5). The f-test is a statistical technique employed to ascertain if there is a significant difference in the means of two groups. The p-value linked to the f-test reflects the probability of obtaining the data if the null hypothesis holds. The null hypothesis is strongly proved by p-value ≤0.05. This suggests that the difference between the groups cannot result from pure chance. Combining both the p and f-test values significantly increases the dependability of the given model. These values allow us to assess the significance of the relationship between properties and indices. A p-value ≤0.05 and f-test value >2.5 are commonly observed indications of a strong and positive association in statistical analysis. Otherwise, if the f-test value is below 2.5, and the p-value exceeds 0.05, it indicates insignificance, indicating a limited link between properties and indices. Evaluating these values ensures model validity and informed decision-making. Table 3, Table 4, Table 5, Table 6, Table 7, Table 8, Table 9, Table 10 comprise the numerical data for 8 parameters, which include regression values, p-values, and f-test values for specified properties of schizophreni and eccentric indices. The results for linear regression for ζ(G) are shown in this Table 3. The correlation value for ζ(G) ranges from 0.0539 to 0.4332, and the p-value varies from 0.8613 to 0.1392. We observe that only validated properties depending on P values are enthalpy, flash point, molar refractivity, and complexity. The values which are not significant p-values are boiling point, melting point, molecular weight, and refractive index. Now, the range of the correlation for the quadratic model varies from 0.1044 to 0.4343, and the p-value varies from 0.9466 to 0.3518. The correlation for this measure with ζ(G) is relatively poor. ζ(G) cannot predict poor values. Only validated p values are enthalpy and complexity. The remaining properties, i.e., boiling point, melting point, flash point, molar refractivity, molecular weight, and refractive index, are insignificant p-values. ζ(G) is a commonly used index in treatment of drugs. The quadratic model has a strong correlation with medicines and drugs. Table 3 shows ζ(G) has the highest correlation with complexity.

**Table 1:** Physicochemical properties of Drugs Used in Schizophrenia Treatment.

drugs	BP	MP	E	FP	MR	C	MW	R
a	450.1	60	70.9	226.0	92.8	339	355.33	93.76
b	506.0	242	77.6	259.8	108.2	510	480.4	110.98
c	515.665	73	78.8	265.7	112.8	432	370.6	113.52
d	599.0	114	89.2	316.1	126.5	711	443.62	137.85
e	529.0	151.5	84.6	273.8	101.0	451	375.9	102.59
f	489.2	183	75.5	249.6	93.7	446	326.8	97.36
g	554.8	213	83.6	289.3	114.1	573	412.936	116.72
h	458.6	109	71.9	231.1	92.1	450	327.81	95.11
i	556.5	172	88.2	290.4	110.2	496	383.51	114.09
j	646.2	139.0	95.3	344.6	120.3	559	448.4	124.34
k	572.4	170.0	85.8	300.0	111.7	731	410.5	111.7
l	476	195	74.0	241.7	92.2	432	312.432	107.17
m	592.1	95	88.3	311.9	120.7	623	440.941	131.77

The statistical data related to average eccentricity avec(G) in Table 4 for linear regression are given in Table 4. The correlation values for properties i.e., BP(0.2119), MP(0.1169), E(0.2375), FP(0.1334), MR(0.0520), C(0.0316), MW(0.0316), and R(0.01), respectively. So, all correlation values are not significant as *r* is less than 0.7, but p values are good only for BP(0.487), E(0.4346), C(0.2993) according to its validity condition. F-test values with highest and lowest significance (1.186,0.0014) with p value (0.2993,0.9703). The correlation values for this model are (14.70,0.01). In the quadratic model, statistical data-related correlation and p values for BP, MP, E, FP, MR, C, MW, and R are not significant because r<0.7 and p values less than equals to 0.05 and f-test value (<2.5). The F-test values with highest and lowest significance (0.8887,0.09321) with p value (0.4413,0.9118). The correlation values for this model are (0.3884,0.1353).

Now, QSPR analysis of $M_{1}(G)$ in Table 5 shows that correlation values for all properties are not significant because r<0.7 and p≤0.05, and f value is significant only for C(2.956) and R(0.25). Also, in the linear model, the highest and lowest f values are (2.956,0.0132) with p values (0.1135,0.9105). and the corresponding correlation values are (0.4602,0.0346). Now, in the quadratic regression model, all correlation, f, and p values are not significant and not valid for the condition. Also, the highest and lowest f values are (0.5537,0.0877) with p values (0.5915,0.9167). and the corresponding correlation values are (0.3158,0.0877).

QSPR analysis for $M_{1}^{⁎}⁎(G)$ in Table 6 shows that correlation and p values are not significant but f value for C(3.937) is significant for linear regression. Also, the highest and lowest f values are (3.937,0.0951) with p values (0.0728,0.7635). and the corresponding correlation values are (0.5134,0.0927). Also, the quadratic regression model is not significant with correlation, f, and p values. Furthermore, the highest and lowest f values are (1.965,0.0459) with p values (0.1906,0.9553). and the corresponding correlation values are (0.5311,0.0954).

QSPR analysis for $M_{2}^{⁎}(G)$ in Table 7 shows that correlation and p-values and f-values are insignificant for linear regression. Also, highest and lowest f values are (1.952,0.0448) with p values (0.1899,0.8362) and the corresponding correlation values are (0.3882,0.0640). Also, the quadratic regression model is insignificant with correlation, f, and p values. Furthermore, highest and lowest f values are (0.9157,0.0614) with p values (0.4313,0.9408) and the corresponding correlation values are (0.3934,0.11).

QSPR analysis for $GA_{4}(G)$ shows in Table 8 that correlation values are not significant for linear regression, but p-value for C(0.01406) is the only significant value and f values at C(8.498) is only a significant value for this linear model. Also, the highest and lowest f values are (8.498,0.0770) with p values (0.01406,0.7865) and the corresponding correlation values are (0.6602,0.0837). Also, the quadratic regression model is not significant with correlation, but only C(0.0431) is a significant p-value, and C(4.376) is the only significant f-value. Furthermore, highest and lowest f values are (4.376,0.6547) with p values (0.0431,0.5405) and the corresponding correlation values are (0.6831,0.3403).

QSPR analysis for ABC(G) in Table 9 shows that correlation values, f, and p values are insignificant for linear regression. Also, highest and lowest f values are (1.809,0.0017) with p values (0.2057,0.9675) and the corresponding correlation values are (0.3758,0.0141). Also, correlation values, f, and p values are insignificant for quadratic regression mode. Also, the highest and lowest f values are (1.57,0.0499) with p values (0.2553,0.9516) and the corresponding correlation values are (0.4887,0.0994).

QSPR analysis for $H_{e}(G)$ in Table 10 shows that except for only one f-value BP(3.687), the correlation values and p values are not significant for linear regression. Also, the highest and lowest f values are (3.687,0.0019) with p values (0.0811,0.96635) and the corresponding correlation values are (0.5010,0.0141). Also, except for only one f-value BP(2.645), the correlation values and p values are not significant for quadratic regression mode. Also, the highest and lowest f values are (2.645,0.2552) with p values (0.1197,0.7796) and the corresponding correlation values are (0.5882,0.2205).

In certain cases, the linear and quadratic regression analyses' comparatively low R-squared values and negligible p-values suggest that more research is necessary. Future research will take into consideration the possibility that including higher-order regression models, like cubic regression, could improve understanding of the connection between eccentric indices and physical characteristics.

When graphically displaying data, line graphs and bar graphs of various lengths are popular and useful methods for displaying data volumes and facilitating comparisons within and across drug groups. The Bar and line graphs show a positive correlation between indices and properties. These graphs also help readers comprehend and analyze data more efficiently. These graphs help to visualize the comparisons of all correlation values. These graphs have been created using Microsoft Excel. The dimensions of graphs depend on the number of variables. The values of correlation lie between 0 and 1. If the r is near one, then it is a very strong positive correlation, and away from one, it is a weaker one. The negative sign with r means two quantities have an inverse relation, see Figure 4, Figure 5, Figure 6, Figure 7, Figure 8, Figure 9, Figure 10, Figure 11.

The eccentric indices are shown on the x-axis, known as the horizontal axis. In contrast, the y-axis, known as the vertical axis, represents the correlation values for all related properties. The length of the bars corresponds to the data being represented. Microsoft Excel was used to create the graphs.

**Figure 3:**
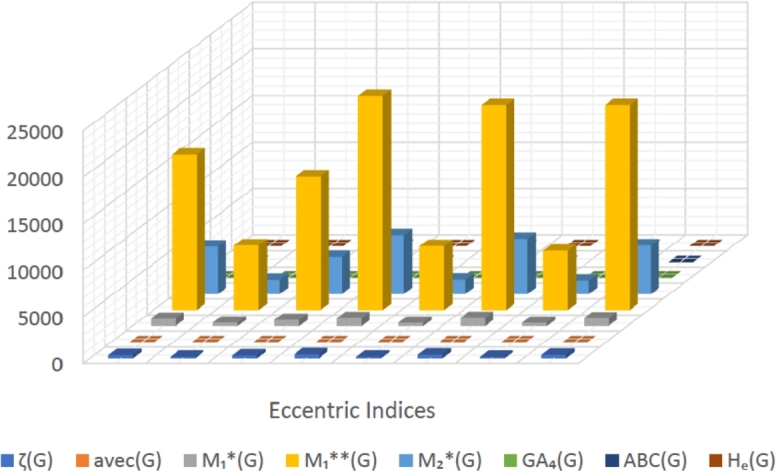
Correlation between EIs and Schizophrenia Drugs.

**Figure 4:**
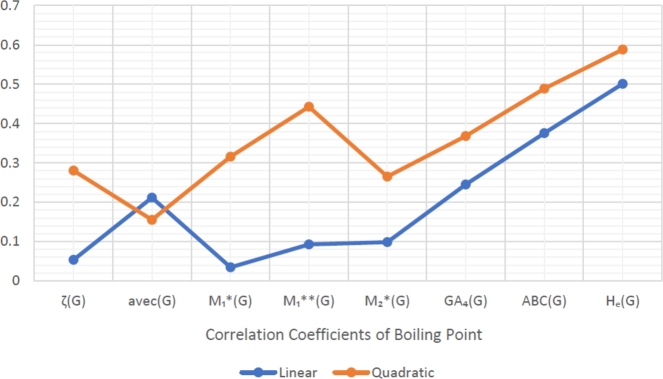
Correlation between EIs and BP.

**Figure 5:**
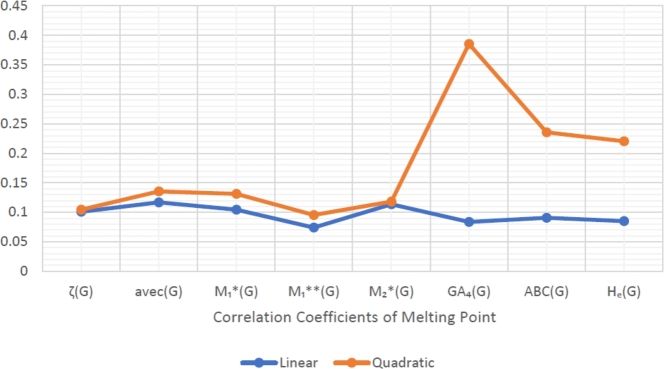
Correlation between EIs and MP.

**Figure 6:**
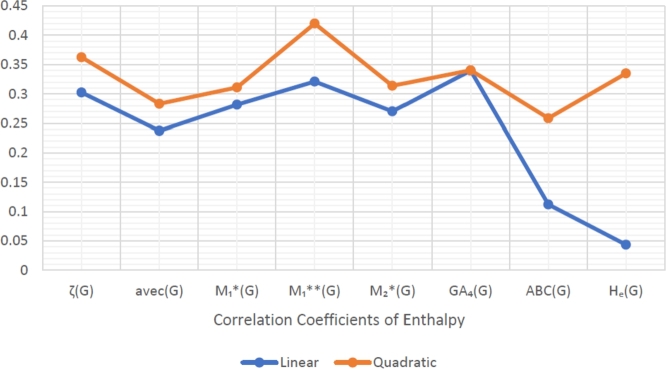
Correlation between EIs and E.

**Figure 7:**
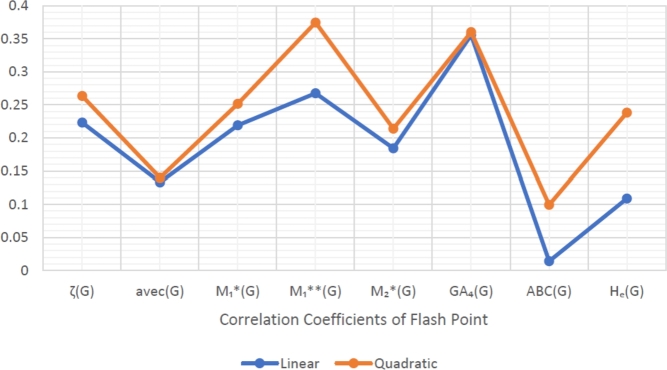
Correlation between EIs and FP.

**Figure 8:**
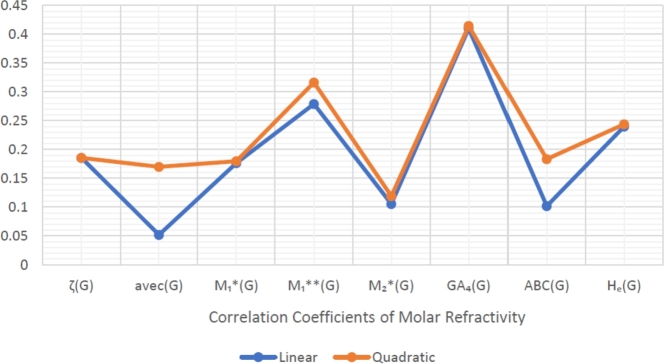
Correlation between EIs and MR.

**Figure 9:**
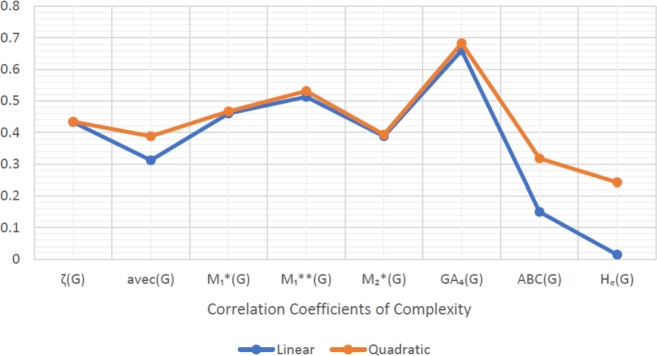
Correlation between EIs and C.

**Figure 10:**
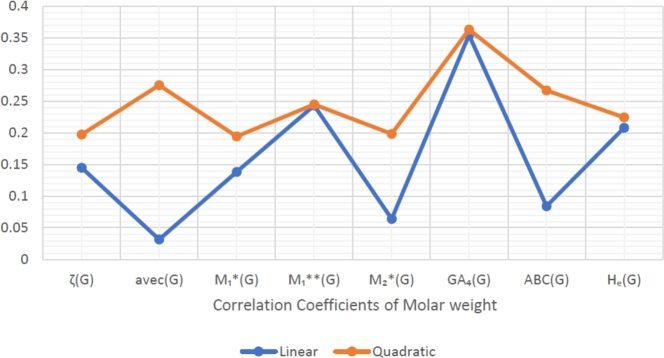
Correlation between EIs and MW.

**Figure 11:**
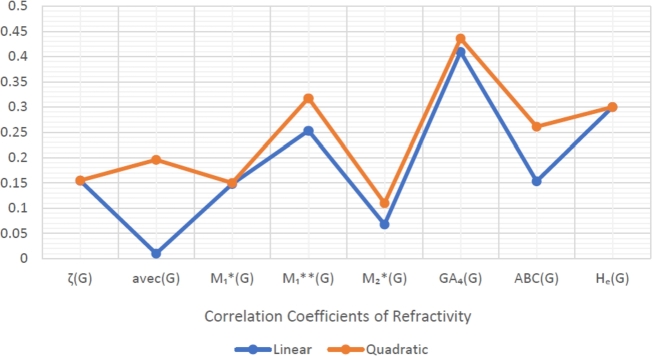
Correlation between EIs and R.

The connection between indices and properties of schizophrenia drugs is shown in Figure 3, Figure 11. Based on information from Table 11, Table 12, Figure 4, Figure 5, Figure 6, Figure 7, Figure 8, Figure 9, Figure 10, Figure 11 provide an understandable representation of the range of *r* values. For example, Figure 4, Figure 5, Figure 6, Figure 7, Figure 8, Figure 9, Figure 10, Figure 11 illustrates the comparison of correlation values with bar graphs of different colors representing the linear and quadratic regression model. The line graph in Fig. 4 shows the positive correlation values between eccentric indices and boiling point BP for the linear and quadratic model lies between 0.0539 and 0.5882. The correlation r results for the linear and quadratic models in Fig. 5 for melting point are all positive and range from 0.0741 to 0.3855. In Fig. 6, The correlation *r* values for enthalpy and eccentric indices for the linear and quadratic models are all positive and range from 0.0741 to 0.3855. Again, in Fig. 7, both linear and quadratic regression models for flash point show a positive correlation with the range from 0.0141 to 0.3748. The correlation r values for molar refractivity range from 0.0520 to 0.4145; see Fig. 8. Fig. 9 shows that the range of correlation values of positive complexity for linear and quadratic models lie between 0.0141 to 0.6831. Now, molecular weight in Fig. 10 shows the range of positive correlation values lie between 0.0316 to 0.3633. In Fig. 11, both linear and quadratic regression models for refractive index show a positive correlation with the range from 0.01 to 0.3748.

We generally observe that both linear and quadratic regression models show positive correlation *r* values ranging from 0.01 to 0.6831 for all eccentric indices and drug properties BP, MP, E, FP, MR, C, *MW*, and *R*. Table 11, Table 12 and Figure 4, Figure 5, Figure 6, Figure 7, Figure 8, Figure 9, Figure 10, Figure 11 illustrate the detailed information for these values. Our primary goal is to discuss QSPR analysis, which focuses on eight different properties, namely BP, MP, E, FP, MR, C, MW, R, and specified eccentric topological indices (EIs) that are related to schizophrenia disease. Table 2 contains the values that were computed for each of the index. The values of the eccentric indices are determined using Table 1. These results were obtained by computing the statistical parameters. The computed values of the statistical parameters are shown in Table 3, Table 10. Every table shows the f-test and p-values for each EIs that are correlated with anti-schizophreni drugs. Certain results, therefore, have strong and significant value. The graphs show a positive and strong correlation and provide a graphical overview of the *r* values between EIs and properties, see Figure 3, Figure 11.

**Table 2:** Thirteen Schizophrenia drugs along with their calculated eccentric indices.

drugs	*ζ*(*G*)	*avec*(*G*)	$M_{1}^{⁎}(G)$	$M_{1}^{⁎⁎}(G)$	$M_{2}^{⁎}(G)$	*GA*_4_(*G*)	*ABC*(*G*)	*H*_*e*_(*G*)
a	156	7.4286	332	5978	1221	22.9450	1.6352	3.2715
b	294	10.5	638	15718	3345	30.9631	1.3187	3.0846
c	218	8.75	478	10238	2074	27.9487	1.5535	3.3510
d	331	11.0333	711	17531	3908	32.9617	1.3154	3.1387
e	339	13.0384	721	14509	4773	27.9782	0.8724	2.2449
f	352	12.5714	799	16738	5139	31.9730	1.0501	2.6557
g	179	7.782	393	7135	1516	25.9401	1.7326	3.5329
h	315	11.6666	691	15261	4099	29.9731	1.1065	2.6988
i	430	14.3333	875	23086	6340	30.9785	0.8447	2.2666
j	180	7.8261	397	6906	1548	25.9413	1.7099	3.4999
k	395	13.1667	887	22057	5946	33.9761	1.0407	2.6923
l	170	7.7272	377	6406	1450	24.9444	1.6689	3.4020
m	383	12.3548	849	22057	5259	34.9685	1.1956	2.9876

**Table 3:** Statistical analysis of *ζ*(*G*) index in QSPR Modeling.

Properties	*n*	*α*	*β*	*γ*	*r*	*r*^2^	*f*	*p*
Linear Regression Model								
BP	13	521.5485	-0.0814	-	0.0539	0.0029	0.0320	0.8613
MP	13	130.6353	0.0583	-	0.1010	0.0102	0.1137	0.7424
E	13	74.9125	0.0240	-	0.3025	0.0915	1.108	0.3151
FP	13	252.9283	0.0834	-	0.2232	0.0498	0.5764	0.4637
MR	13	100.7184	0.0232	-	0.1857	0.0345	0.3935	0.5433
C	13	368.884	0.5231	-	0.4332	0.1877	2.542	0.1392
MW	13	368.395	0.0801	-	0.1449	0.0210	0.2355	0.637
R	13	105.7800	0.0219	-	0.1549	0.0240	0.2706	0.6133
Quadratic Regression Model								
BP	13	957.4750	-3.5467	0.0062	0.2804	0.0786	0.4263	0.6642
MP	13	114.7	0.1853	-0.0002	0.1044	0.0109	0.0552	0.9466
E	13	91.5359	-0.1081	0.0002	0.3625	0.1314	0.7564	0.4944
FP	13	307.9	-0.3538	0.0008	0.2636	0.0695	0.3735	0.6975
MR	13	100.6	0.0244	-0.0000	0.1857	0.0345	0.1789	0.8388
C	13	331.8	0.8175	-0.0005	0.4343	0.1886	1.162	0.3518
MW	13	290.4674	0.6996	-0.0011	0.1975	0.0390	0.2027	0.8198
R	13	107.6	0.0076	0.0000	0.1552	0.02415	0.1238	0.8849

**Table 4:** Statistical analysis of *avec*(*G*) index in QSPR Modeling.

Properties	*n*	*α*	*β*	*γ*	*r*	*r*^2^	*f*	*p*
Linear Regression Model								
BP	13	630.91	-12.49	-	0.2119	0.04491	0.5172	0.487
MP	13	119.470	2.630	-	0.1169	0.01367	0.1524	0.7037
E	13	74.0074	0.7353	-	0.2375	0.05642	0.6577	0.4346
FP	13	256.278	1.942	-	0.1334	0.0178	0.199	0.6642
MR	13	104.6725	0.2523	-	0.0520	0.0027	0.0298	0.866
C	13	363.26	14.70	-	0.3121	0.0974	1.186	0.2993
MW	13	384.3785	0.6677	-	0.0316	0.0010	0.0105	0.9202
R	13	111.4023	0.0632	-	0.01	0.0001	0.0014	0.9703
Quadratic Regression Model								
BP	13	107.6	0.0076	0.0000	0.1554	0.02415	0.1238	0.8849
MP	13	216.2189	-16.7138	0.9184	0.1353	0.0183	0.09321	0.9118
E	13	104.1575	-5.2928	0.2862	0.2831	0.08017	0.4358	0.6585
FP	13	295.6698	-5.2928	0.3739	0.14	0.0196	0.0999	0.9058
MR	13	55.0361	10.1764	-0.4712	0.1701	0.02892	0.1489	0.8635
C	13	-325.515	152.407	-6.538	0.3884	0.1509	0.8887	0.4413
MW	13	10.582	75.403	-3.548	0.2755	0.07592	0.4108	0.6738
R	13	43.3157	13.6762	-0.6463	0.1962	0.0385	0.1999	0.822

**Table 5:** Statistical analysis of *M*_1_(*G*) index in QSPR Modeling.

Properties	*n*	*α*	*β*	*γ*	*r*	*r*^2^	*f*	*p*
Linear Regression Model								
BP	13	513.3930	-0.0244	-	0.0346	0.0012	0.0132	0.9105
MP	13	129.9090	0.0279	-	0.1044	0.0109	0.1207	0.7348
E	13	75.3042	0.0104	-	0.2818	0.0794	0.9489	0.3509
FP	13	253.0580	0.0381	-	0.2191	0.0480	0.5551	0.4719
MR	13	100.9745	0.0103	-	0.1766	0.0312	0.3537	0.5641
C	13	357.4807	0.2584	-	0.4602	0.2118	2.956	0.1135
MW	13	369.1599	0.0356	-	0.1382	0.0191	0.2143	0.6525
R	13	106.0	0.0097	-	0.1480	0.0219	0.246	0.6297
Quadratic Regression Model								
BP	13	1060	-2.034	0.0017	0.3158	0.0997	0.5537	0.5915
MP	13	76.89	0.2230	-0.0002	0.1311	0.0172	0.0877	0.9167
E	13	87.32	-0.0338	0.0000	0.3110	0.0967	0.5353	0.6014
FP	13	306.4	-0.1580	0.0002	0.2518	0.0634	0.3385	0.7207
MR	13	96.14	0.0281	-0.000	0.1797	0.0323	0.1668	0.8487
C	13	468.8	-0.1511	0.0003	0.4671	0.2182	1.396	0.292
MW	13	281.9	0.3565	-0.000	0.1944	0.0378	0.1965	0.8247
R	13	101.7	0.0256	-0.000	0.1503	0.02258	0.1155	0.8921

**Table 6:** Statistical analysis of $M_{1}^{⁎⁎}(G)$ index in QSPR Modeling.

Properties	*n*	*α*	*β*	*γ*	*r*	*r*^2^	*f*	*p*
Linear Regression Model								
BP	13	467.9	0.0021	-	0.0927	0.0086	0.0951	0.7635
MP	13	138.2	0.0007	-	0.0741	0.0055	0.0611	0.8094
E	13	76.33	0.0004	-	0.3210	0.1031	1.265	0.2847
FP	13	255.4	0.0015	-	0.2676	0.0716	0.8477	0.377
MR	13	99.88	0.0005	-	0.2789	0.0778	0.9285	0.356
C	13	385.7	0.0095	-	0.5134	0.2636	3.937	0.0728
MW	13	362.4	0.0021	-	0.2433	0.0592	0.6924	0.423
R	13	104.4	0.0005	-	0.2528	0.0639	0.7513	0.4046
Quadratic Regression Model								
BP	13	808.6	-0.0568	0.0000	0.4426	0.1959	1.218	0.3363
MP	13	120.2	0.0038	-0.0000	0.0954	0.0091	0.0459	0.9553
E	13	87.52	-0.0015	0.0000	0.42	0.1764	1.071	0.3789
FP	13	306.4	-0.0073	0.0000	0.3748	0.1405	0.8171	0.4691
MR	13	109.6	-0.0011	-0.0000	0.3164	0.1001	0.5561	0.5902
C	13	471.5	-0.0053	0.0000	0.5311	0.2821	1.965	0.1906
MW	13	370.8	0.0006	0.0000	0.2451	0.0601	0.3196	0.7336
R	13	118.4	-0.0019	0.0000	0.3169	0.1004	0.5582	0.5891

**Table 7:** Statistical analysis of $M_{2}^{⁎}(G)$ index in QSPR Modeling.

Properties	*n*	*α*	*β*	*γ*	*r*	*r*^2^	*f*	*p*
Linear Regression Model								
BP	13	525.7316	-0.0077	-	0.0985	0.0097	0.1079	0.7487
MP	13	135.3	0.0033	-	0.1136	0.0129	0.144	0.7116
E	13	77.8483	0.0011	-	0.2702	0.0730	0.8663	0.372
FP	13	264.2	0.0036	-	0.1844	0.0340	0.3873	0.5464
MR	13	105.0	0.0007	-	0.1054	0.0111	0.1239	0.7315
C	13	432.5803	0.0242	-	0.3882	0.1507	1.952	0.1899
MW	13	384.9	0.0018	-	0.0640	0.0041	0.0448	0.8362
R	13	110.3	0.0005	-	0.0678	0.0046	0.0510	0.8254
Quadratic Regression Model								
BP	13	672.1	-0.1156	0.0000	0.2653	0.0704	0.3785	0.6943
MP	13	142.7	-0.0021	0.0000	0.1183	0.0140	0.0709	0.932
E	13	82.84	-0.0025	0.0000	0.3140	0.0986	0.5469	0.5951
FP	13	280.1	-0.008213	0.0000	0.2140	0.0458	0.2402	0.7909
MR	13	102.3	0.0027	-0.0000	0.1192	0.0142	0.0718	0.9312
C	13	402.2	0.0466	-0.0000	0.3934	0.1548	0.9157	0.4313
MW	13	344.0	0.0320	-0.0000	0.1985	0.0394	0.2051	0.8179
R	13	105.5	0.0040	-0.0000	0.11	0.0121	0.0614	0.9408

**Table 8:** Statistical analysis of *GA*_4_(*G*) index in QSPR Modeling.

Properties	*n*	*α*	*β*	*γ*	*r*	*r*^2^	*f*	*p*
Linear Regression Model								
BP	13	218.812	9.518	-	0.2449	0.06	0.7022	0.4199
MP	13	111.145	1.236	-	0.0837	0.0070	0.0770	0.7865
E	13	61.4845	0.6931	-	0.3397	0.1154	1.435	0.2561
FP	13	176.666	3.416	-	0.3558	0.1266	1.594	0.2329
MR	13	68.7324	1.3179	-	0.41	0.1681	2.222	0.1641
C	13	-81.90	20.49	-	0.6602	0.4358	8.498	0.01406
MW	13	243.287	5.050	-	0.3547	0.1258	1.583	0.2344
R	13	68.493	1.485	-	0.4094	0.1676	2.214	0.1648
Quadratic Regression Model								
BP	13	2879.580	-176.143	3.190	0.3682	0.1356	0.784	0.4827
MP	13	-1278.924	98.231	-1.666	0.3855	0.1486	0.8726	0.4474
E	13	51.4793	1.3912	-0.0120	0.3403	0.1158	0.6547	0.5405
FP	13	312.7219	-6.0771	0.1631	0.3603	0.1298	0.7458	0.499
MR	13	117.6977	-2.0987	0.0587	0.4145	0.1718	1.037	0.3896
C	13	1276.795	-74.313	1.629	0.6831	0.4667	4.376	0.0431
MW	13	523.9205	-14.5320	0.3364	0.3633	0.132	0.7606	0.4926
R	13	203.4783	-7.9338	0.1618	0.4358	0.1899	1.172	0.3489

**Table 9:** Statistical analysis of *ABC*(*G*) index in QSPR Modeling.

Properties	*n*	*α*	*β*	*γ*	*r*	*r*^2^	*f*	*p*
Linear Regression Model								
BP	13	276.1	169.3	-	0.3758	0.1412	1.809	0.2057
MP	13	167.83	-15.56	-	0.0906	0.0082	0.0909	0.7687
E	13	85.310	-2.659	-	0.1122	0.0126	0.1407	0.7147
FP	13	275.09	1.40	-	0.0141	0.0002	0.0017	0.9675
MR	13	102.414	3.809	-	0.1020	0.0104	0.1161	0.7398
C	13	590.3	-54.0	-	0.1500	0.0225	0.2533	0.6247
MW	13	373.23	13.92	-	0.0843	0.0071	0.0787	0.7843
R	13	103.612	6.454	-	0.1533	0.0235	0.2651	0.6168
Quadratic Regression Model								
BP	13	-707.6	1758.4	-606.2	0.4887	0.2389	1.57	0.2553
MP	13	429.0	-437.4	160.9	0.2355	0.0555	0.2938	0.7516
E	13	123.81	-64.86	23.73	0.2586	0.0669	0.3587	0.7072
FP	13	351.77	-122.47	47.26	0.0994	0.0099	0.0499	0.9516
MR	13	62.75	67.87	-24.44	0.1835	0.0337	0.1741	0.8427
C	13	-112.9	1081.9	-433.3	0.3175	0.1008	0.5603	0.588
MW	13	81.06	485.85	-180.04	0.2670	0.0713	0.3839	0.6908
R	13	41.64	106.55	-38.19	0.2608	0.0680	0.365	0.7031

**Table 10:** Statistical analysis of *H*_*e*_(*G*) index in QSPR Modeling.

Properties	*n*	*α*	*β*	*γ*	*r*	*r*^2^	*f*	*p*
Linear Regression Model								
BP	13	7.104	164.403	-	0.5010	0.251	3.687	0.0811
MP	13	179.11	-10.61	-	0.0849	0.0072	0.0796	0.7831
E	13	84.0464	-0.7444	-	0.0436	0.0019	0.0206	0.8885
FP	13	250.454	8.862	-	0.1091	0.0119	0.133	0.7223
MR	13	87.945	6.517	-	0.2400	0.0576	0.6726	0.4295
C	13	509.265	3.414	-	0.0141	0.0002	0.0019	0.9663
MW	13	316.69	25.04	-	0.2081	0.0433	0.4987	0.4948
R	13	84.648	9.183	-	0.2997	0.0898	1.086	0.3198
Quadratic Regression Model								
BP	13	-2238.0	1750.2	-273.8	0.5882	0.346	2.645	0.1197
MP	13	744.67	-410.08	68.96	0.2205	0.0486	0.2552	0.7796
E	13	211.18	-90.54	15.50	0.3351	0.1123	0.6324	0.5513
FP	13	632.00	-260.64	46.53	0.2383	0.0568	0.3013	0.7463
MR	13	114.248	-12.062	3.207	0.2439	0.0595	0.3165	0.7357
C	13	-897.5	997.0	-171.5	0.2421	0.0586	0.3111	0.7395
MW	13	90.92	184.51	-27.53	0.2247	0.0505	0.266	0.7717
R	13	77.1381	14.4873	-0.9158	0.2998	0.0899	0.4943	0.6241

**Table 11:** Table of comparisons for linear regression correlated coefficient.

Indices	BP	MP	E	FP	MR	C	MW	R
*ζ*(*G*)	0.0539	0.1010	0.3025	0.2232	0.1857	0.4332	0.1449	0.1549
*avec*(*G*)	0.2119	0.1169	0.2375	0.1334	0.0520	0.3121	0.0316	0.01
$M_{1}^{⁎}(G)$	0.0346	0.1044	0.2818	0.2191	0.1766	0.4602	0.1382	0.1480
$M_{1}^{⁎⁎}(G)$	0.0927	0.0741	0.3210	0.2676	0.2789	0.5134	0.2433	0.2528
$M_{2}^{⁎}(G)$	0.0985	0.1136	0.2702	0.1844	0.1054	0.3882	0.0640	0.0678
*GA*_4_(*G*)	0.2449	0.0837	0.3397	0.3558	0.41	0.6602	0.3547	0.4094
*ABC*(*G*)	0.3758	0.0906	0.1122	0.0141	0.1020	0.1500	0.0843	0.1533
*H*_*e*_(*G*)	0.5010	0.0849	0.0436	0.1091	0.2400	0.0141	0.2081	0.2997

**Table 12:** Table of comparisons for quadratic regression correlated coefficient.

Indices	BP	MP	E	FP	MR	C	MW	R
*ζ*(*G*)	0.2804	0.1044	0.3625	0.2636	0.1857	0.4343	0.1975	0.1552
*avec*(*G*)	0.1554	0.1353	0.2831	0.14	0.1701	0.3884	0.2755	0.1962
$M_{1}^{⁎}(G)$	0.3158	0.1311	0.3110	0.2518	0.1797	0.4671	0.1944	0.1503
$M_{1}^{⁎}⁎(G)$	0.4426	0.0954	0.42	0.3748	0.3164	0.5311	0.2451	0.3169
$M_{2}^{⁎}(G)$	0.2653	0.1183	0.3140	0.2140	0.1192	0.3934	0.1985	0.11
*GA*_4_(*G*)	0.3682	0.3855	0.3403	0.3603	0.4145	0.6831	0.3633	0.4358
*ABC*(*G*)	0.4887	0.2355	0.2586	0.0994	0.1835	0.3175	0.2670	0.2608
*H*_*e*_(*G*)	0.5882	0.2205	0.3351	0.2383	0.2439	0.2421	0.2247	0.2998

## 4 Regression models for total eccentric index ζ(G)

$\begin{array}{cc} & BP=521.5485-0.0814(\zeta (G))\\ & MP=130.6353+0.0583(\zeta (G))\\ & E=74.9125+0.0240(\zeta (G))\\ & FP=252.9283+0.0834(\zeta (G))\\ & MR=100.7184+0.0232(\zeta (G))\\ & C=368.884+0.5231(\zeta (G))\\ & MW=368.395+0.0801(\zeta (G))\\ & R=105.7800+0.0219(\zeta (G)) \end{array}\begin{array}{cc} & BP=957.4750{\left(\zeta (G)\right)}^{2}-3.5467(\zeta (G))+0.0062\\ & MP=114.7{\left(\zeta (G)\right)}^{2}+0.1853(\zeta (G))-0.0002\\ & E=91.5359{\left(\zeta (G)\right)}^{2}-0.1081(\zeta (G))+0.0002\\ & FP=307.9{\left(\zeta (G)\right)}^{2}-0.3538(\zeta (G))+0.0008\\ & MR=100.6{\left(\zeta (G)\right)}^{2}+0.0244(\zeta (G))-0.0000\\ & C=331.8{\left(\zeta (G)\right)}^{2}+0.8175(\zeta (G))--0.0005\\ & MW=290.4674{\left(\zeta (G)\right)}^{2}+0.6996(\zeta (G))-0.0011\\ & R=107.6{\left(\zeta (G)\right)}^{2}+0.0076(\zeta (G))+0.0000 \end{array}$

## 5 Regression models for total eccentric index avec(G)

$$\begin{array}{cc} & BP=630.91-12.49(avec(G))\\ & MP=119.470+2.630(avec(G))\\ & E=74.0074+0.7353(avec(G))\\ & FP=256.278+1.942(avec(G))\\ & MR=104.6725+0.2523(avec(G))\\ & C=363.26+14.70(avec(G))\\ & MW=384.3785+0.6677(avec(G))\\ & R=111.4023+0.0632(avec(G)) \end{array}\begin{array}{cc} & BP=107.6{\left(avec(G)\right)}^{2}+0.0076(avec(G))+0.0000\\ & MP=216.2189{\left(avec(G)\right)}^{2}-16.7138(avec(G))+0.9184\\ & E=104.1575{\left(avec(G)\right)}^{2}-5.2928(avec(G))+0.2862\\ & FP=295.6698{\left(avec(G)\right)}^{2}--5.2928(avec(G))+0.3739\\ & MR=55.0361{\left(avec(G)\right)}^{2}+10.1764(avec(G))-0.4712\\ & C=-325.515{\left(avec(G)\right)}^{2}+152.407(avec(G))-6.538\\ & MW=10.582{\left(avec(G)\right)}^{2}+75.403(avec(G))-3.548\\ & R=43.3157{\left(avec(G)\right)}^{2}+13.6762(avec(G))-0.6463 \end{array}$$

## 6 Regression models for total eccentric index $M_{1}^{⁎}(G)$

$$\begin{array}{cc} & BP=513.3930-0.0244(M_{1}^{⁎}(G))\\ & MP=129.9090+0.0279(M_{1}^{⁎}(G))\\ & E=75.3042+0.0104(M_{1}^{⁎}(G))\\ & FP=253.0580+0.0381(M_{1}^{⁎}(G))\\ & MR=100.9745+0.0103(M_{1}^{⁎}(G))\\ & C=357.4807+0.2584(M_{1}^{⁎}(G))\\ & MW=369.1599+0.0356(M_{1}^{⁎}(G))\\ & R=106.0+0.0097(M_{1}^{⁎}(G)) \end{array}\begin{array}{cc} & BP=1060{\left(M_{1}^{⁎}(G)\right)}^{2}-2.034(M_{1}^{⁎}(G))+0.0017\\ & MP=76.89{\left(M_{1}^{⁎}(G)\right)}^{2}+0.2230(M_{1}^{⁎}(G))-0.0002\\ & E=87.32{\left(M_{1}^{⁎}(G)\right)}^{2}-0.0338(M_{1}^{⁎}(G))+0.0000\\ & FP=306.4{\left(M_{1}^{⁎}(G)\right)}^{2}-0.1580(M_{1}^{⁎}(G))=0.0002\\ & MR=96.14{\left(M_{1}^{⁎}(G)\right)}^{2}+0.0281(M_{1}^{⁎}(G))--0.000\\ & C=468.8{\left(M_{1}^{⁎}(G)\right)}^{2}-0.1511(M_{1}^{⁎}(G))+0.0003\\ & MW=281.9{\left(M_{1}^{⁎}(G)\right)}^{2}+0.3565(M_{1}^{⁎}(G))-0.000\\ & R=101.7{\left(M_{1}^{⁎}(G)\right)}^{2}+0.0256(M_{1}^{⁎}(G))-0.000 \end{array}$$

## 7 Regression models for total eccentric index $M_{1}^{⁎⁎}(G)$

$$\begin{array}{cc} & BP=467.9+0.0021(M_{1}^{⁎⁎}(G))\\ & MP=138.2+0.0007(M_{1}^{⁎⁎}(G))\\ & E=76.33+0.0004(M_{1}^{⁎⁎}(G))\\ & FP=255.4+0.0015(M_{1}^{⁎⁎}(G))\\ & MR=99.88+0.0005(M_{1}^{⁎⁎}(G))\\ & C=385.7+0.0095(M_{1}^{⁎⁎}(G))\\ & MW=362.4+0.0021(M_{1}^{⁎⁎}(G))\\ & R=104.4+0.0005(M_{1}^{⁎⁎}(G)) \end{array}\begin{array}{cc} & BP=808.6{\left(M_{1}^{⁎⁎}(G)\right)}^{2}-0.0568(M_{1}^{⁎⁎}(G))+0.0000\\ & MP=120.2{\left(M_{1}^{⁎⁎}(G)\right)}^{2}+0.0038(M_{1}^{⁎⁎}(G))-0.0000\\ & E=87.52{\left(M_{1}^{⁎⁎}(G)\right)}^{2}-0.0015(M_{1}^{⁎⁎}(G))+0.0000\\ & FP=306.4{\left(M_{1}^{⁎⁎}(G)\right)}^{2}-0.0073(M_{1}^{⁎⁎}(G))+0.0000\\ & MR=109.6{\left(M_{1}^{⁎⁎}(G)\right)}^{2}-0.0011(M_{1}^{⁎⁎}(G))-0.0000\\ & C=471.5{\left(M_{1}^{⁎⁎}(G)\right)}^{2}-0.0053(M_{1}^{⁎⁎}(G))+0.0000\\ & MW=370.8{\left(M_{1}^{⁎⁎}(G)\right)}^{2}+0.0006(M_{1}^{⁎⁎})+0.0000\\ & R=118.4{\left(M_{1}^{⁎⁎}(G)\right)}^{2}-0.0019(M_{1}^{⁎⁎}(G))+0.0000 \end{array}$$

## 8 Regression models for total eccentric index $M_{2}^{⁎}(G)$

$$\begin{array}{cc} & BP=525.7316-0.0077(M_{2}^{⁎}(G))\\ & MP=135.3+0.0033(M_{2}^{⁎}(G))\\ & E=77.8483+0.0011(M_{2}^{⁎}(G))\\ & FP=264.2+0.0036(M_{2}^{⁎}(G))\\ & MR=105.0+0.0007(M_{2}^{⁎}(G))\\ & C=432.5803+0.0242(M_{2}^{⁎}(G))\\ & MW=384.9+0.0018(M_{2}^{⁎}(G))\\ & R=110.3+0.0005(M_{2}^{⁎}(G)) \end{array}\begin{array}{cc} & BP=672.1{\left(M_{2}^{⁎}(G)\right)}^{2}-0.1156(M_{2}^{⁎}(G))+0.0000\\ & MP=142.7{\left(M_{2}^{⁎}(G)\right)}^{2}-0.0021(M_{2}^{⁎}(G))+0.0000\\ & E=82.84{\left(M_{2}^{⁎}(G)\right)}^{2}-0.0025(M_{2}^{⁎}(G))+0.0000\\ & FP=280.1{\left(M_{2}^{⁎}(G)\right)}^{2}-0.008213(M_{2}^{⁎}(G))+0.0000\\ & MR=102.3{\left(M_{2}^{⁎}(G)\right)}^{2}+0.0027(M_{2}^{⁎}(G))-0.0000\\ & C=402.2{\left(M_{2}^{⁎}(G)\right)}^{2}+0.0466(M_{2}^{⁎}(G))-0.0000\\ & MW=344.0{\left(M_{2}^{⁎}(G)\right)}^{2}+0.0320(M_{2}^{⁎}(G))-0.0000\\ & R=105.5{\left(M_{2}^{⁎}(G)\right)}^{2}+0.0040(M_{2}^{⁎}(G))-0.0000 \end{array}$$

## 9 Regression models for total eccentric index $GA_{4}(G)$

$$\begin{array}{cc} & BP=218.812+9.518(GA_{4}(G))\\ & MP=111.145+1.236(GA_{4}(G))\\ & E=61.4845+0.6931(GA_{4}(G))\\ & FP=176.666+3.416(GA_{4}(G))\\ & MR=68.7324+1.3179(GA_{4}(G))\\ & C=-81.90+20.49(GA_{4}(G))\\ & MW=243.287+5.050(GA_{4}(G))\\ & R=68.493+1.485(GA_{4}(G)) \end{array}\begin{array}{cc} & BP=2879.580{\left(GA_{4}(G)\right)}^{2}-176.143(M_{2}^{⁎}(G))+3.190\\ & MP=-1278.924{\left(GA_{4}(G)\right)}^{2}+98.231(M_{2}^{⁎}(G))-1.666\\ & E=51.4793{\left(GA_{4}(G)\right)}^{2}+1.3912(M_{2}^{⁎}(G))-0.0120\\ & FP=312.7219{\left(GA_{4}(G)\right)}^{2}-6.0771(M_{2}^{⁎}(G))+0.1631\\ & MR=117.6977{\left(GA_{4}(G)\right)}^{2}+-2.0987(M_{2}^{⁎}(G))+0.0587\\ & C=1276.795{\left(GA_{4}(G)\right)}^{2}+-74.313(M_{2}^{⁎}(G))+1.629\\ & MW=523.9205{\left(GA_{4}(G)\right)}^{2}+-14.5320(M_{2}^{⁎}(G))+0.3364\\ & R=203.4783{\left(GA_{4}(G)\right)}^{2}+-7.9338(M_{2}^{⁎}(G))+0.1618 \end{array}$$

## 10 Regression models for total eccentric index ABC(G)

$$\begin{array}{cc} & BP=276.1+169.3(ABC(G))\\ & MP=167.83-15.56(ABC(G))\\ & E=85.310-2.659(ABC(G))\\ & FP=275.09+1.40(ABC(G))\\ & MR=102.414+3.809(ABC(G))\\ & C=590.3-54.0(ABC(G))\\ & MW=373.23+13.92(ABC(G))\\ & R=103.612+6.454(ABC(G)) \end{array}\begin{array}{cc} & BP=-707.6{\left(ABC(G)\right)}^{2}+1758.4(ABC(G))-606.2\\ & MP=429.0{\left(ABC(G)\right)}^{2}--437.4(ABC(G))+160.9\\ & E=123.81{\left(ABC(G)\right)}^{2}--64.86(ABC(G))+23.73\\ & FP=351.77{\left(ABC(G)\right)}^{2}-122.47(ABC(G))+47.26\\ & MR=62.75{\left(ABC(G)\right)}^{2}+67.87(ABC(G))-24.44\\ & C=-112.9{\left(ABC(G)\right)}^{2}+1081.9(ABC(G))-433.3\\ & MW=81.06{\left(ABC(G)\right)}^{2}+485.85(ABC(G))-180.04\\ & R=41.64{\left(ABC(G)\right)}^{2}+106.55(ABC(G))-38.19 \end{array}$$

## 11 Regression models for total eccentric index $H_{e}(G)$

$$\begin{array}{cc} & BP=7.104+164.403(H_{e}(G))\\ & MP=179.11-10.61(H_{e}(G))\\ & E=84.0464-0.7444(H_{e}(G))\\ & FP=250.454+8.862(H_{e}(G))\\ & MR=87.945+6.517(H_{e}(G))\\ & C=509.265+3.414(H_{e}(G))\\ & MW=316.69+25.04(H_{e}(G))\\ & R=84.648+9.183(H_{e}(G)) \end{array}\begin{array}{cc} & BP=-2238.0{\left(H_{e}(G)\right)}^{2}+1750.2(H_{e}(G))-273.8\\ & MP=744.67{\left(H_{e}(G)\right)}^{2}-410.08(H_{e}(G))+68.96\\ & E=211.18{\left(H_{e}(G)\right)}^{2}-90.54(H_{e}(G))+15.50\\ & FP=632.00{\left(H_{e}(G)\right)}^{2}-260.64(H_{e}(G))+46.53\\ & MR=114.248{\left(H_{e}(G)\right)}^{2}-12.062(H_{e}(G))+3.207\\ & C=-897.5{\left(H_{e}(G)\right)}^{2}+997.0(H_{e}(G))-171.5\\ & MW=90.92{\left(H_{e}(G)\right)}^{2}+184.51(H_{e}(G))-27.53\\ & R=77.1381{\left(H_{e}(G)\right)}^{2}+14.4873(H_{e}(G))-0.9158 \end{array}$$

## 12 Conclusion

When analyzing drugs using QSPR analysis, eccentric indices are important to predict the drug properties with molecular descriptors. The drugs mentioned above have been extensively utilized in treating schizophrenia. This study is crucial for the development of successful treatment. The eccentricity indices perform similarly well in capturing molecular complexity, especially when it comes to their correlations with the physical characteristics of drugs used to treat schizophrenia. In addition, we analyze these 2 regression models to find out how these computed values relate to calculated values and actual properties such as BP, MP, E, FP, MR, C, MW, and R. We find that for the linear model, Complexity has a highly substantial correlation for ξ(G), avec(G), $M_{1}^{⁎}(G)$, $M_{1}^{⁎⁎}(G)$, $M_{2}^{⁎}(G)$, $GA_{4}(G)$ at r=0.4332, 0.3121, 0.4602, 0.5134, 0.3882, 0.6602, respectively, and also for quadratic regression at r=0.4343, 0.3884, 0.4671, 0.5311, 0.3934, 0.6831, 0.3175. For drugs used to treat schizophrenia, eccentric indices of chemical structures are calculated and correlated using linear and quadratic QSPR models, and it was found that only C is significant, fits, and has a strong correlation in both linear and quadratic correlation models.

## CRediT authorship contribution statement

**Muneeba Mansha:** Writing – original draft, Validation, Software, Methodology, Investigation, Formal analysis, Data curation. **Sarfraz Ahmad:** Writing – review & editing, Supervision, Resources, Project administration, Methodology, Investigation, Formal analysis, Conceptualization. **Zahid Raza:** Writing – review & editing, Supervision, Project administration, Formal analysis, Conceptualization.

## Declaration of Competing Interest

The authors declare the following financial interests/personal relationships which may be considered as potential competing interests: Zahid Raza is the Associate Editor for Heliyon. If there are other authors, they declare that they have no known competing financial interests or personal relationships that could have appeared to influence the work reported in this paper.

## Data Availability

No Data is associated with the manuscript.
